# A Mouse Strain Where Basal Connective Tissue Growth Factor Gene Expression Can Be Switched from Low to High

**DOI:** 10.1371/journal.pone.0012909

**Published:** 2010-09-22

**Authors:** Heather E. Doherty, Hyung-Suk Kim, Sylvia Hiller, Kathleen K. Sulik, Nobuyo Maeda

**Affiliations:** 1 Curriculum in Genetics and Molecular Biology, University of North Carolina at Chapel Hill, Chapel Hill, North Carolina, United States of America; 2 Department of Pathology and Laboratory Medicine, School of Medicine, University of North Carolina at Chapel Hill, Chapel Hill, North Carolina, United States of America; 3 Department of Cell and Developmental Biology, University of North Carolina at Chapel Hill, Chapel Hill, North Carolina, United States of America; 4 Bowles Center for Alcohol Studies, University of North Carolina at Chapel Hill, Chapel Hill, North Carolina, United States of America; The University of Hong Kong, China

## Abstract

Connective tissue growth factor (CTGF) is a signaling molecule that primarily functions in extracellular matrix maintenance and repair. Increased *Ctgf* expression is associated with fibrosis in chronic organ injury. Studying the role of CTGF in fibrotic disease *in vivo*, however, has been hampered by perinatal lethality of the *Ctgf* null mice as well as the limited scope of previous mouse models of *Ctgf* overproduction. Here, we devised a new approach and engineered a single mutant mouse strain where the endogenous *Ctgf*-3′ untranslated region (3′UTR) was replaced with a cassette containing two 3′UTR sequences arranged in tandem. The modified *Ctgf* allele uses a 3′UTR from the mouse FBJ osteosarcoma oncogene (*c-Fos*) and produces an unstable mRNA, resulting in 60% of normal *Ctgf* expression (Lo allele). Upon Cre-expression, excision of the *c-Fos*-3′UTR creates a transcript utilizing the more stable bovine growth hormone (*bGH*) 3′UTR, resulting in increased *Ctgf* expression (Hi allele). Using the *Ctgf* Lo and Hi mutants, and crosses to a *Ctgf* knockout or Cre-expressing mice, we have generated a series of strains with a 30-fold range of *Ctgf* expression. Mice with the lowest *Ctgf* expression, 30% of normal, appear healthy, while a global nine-fold overexpression of *Ctgf* causes abnormalities, including developmental delay and craniofacial defects, and embryonic death at E10-12. Overexpression of *Ctgf* by tamoxifen-inducible Cre in the postnatal life, on the other hand, is compatible with life. The *Ctgf* Lo-Hi mutant mice should prove useful in further understanding the function of CTGF in fibrotic diseases. Additionally, this method can be used for the production of mouse lines with quantitative variations in other genes, particularly with genes that are broadly expressed, have distinct functions in different tissues, or where altered gene expression is not compatible with normal development.

## Introduction

Many of the diseases that have a major impact on human health and pose a major burden on healthcare costs have a fibrosis-related component. Coronary heart disease (CHD) was the single largest killer in the United States in 2003, accounting for 1 in every 5 deaths [1]. Forty percent of acute CHD incidents result in death, but for those who survive an ischemic event, permanent scarring of heart tissue is largely unavoidable. Both myocardial infraction (MI) and chronic high blood pressure result in scarring of the myocardium known as cardiac fibrosis [2]. Cardiac fibrosis is characterized by necrosis of myocardial tissue, collagen buildup, and scar tissue contraction often resulting in ventricular diastolic dysfunction and a poor long term prognosis [3]–[5]. Similarly, both diabetic nephropathy and cirrhosis of the liver have been identified as diseases with a large burden on the healthcare system and are characterized by fibrotic tissue damage [6], [7]. Prevention of cardiac fibrosis and fibrotic disease in other organs (kidney, liver, lung and others) is a promising strategy of intervention for improving long term prognosis and quality of life.

All of the fibrosis-related diseases mentioned are characterized by overexpression of connective tissue growth factor (CTGF or CCN2, ENSMUSG00000019997), a primary fibrosis-related signaling molecule [8]. CTGF is a member of the CCN (Cyr61, Ctgf, and Nov) family of extracellular matrix (ECM) associated proteins. *Ctgf* is widely expressed during development, and in adulthood it is expressed in all major organs including the vasculature and the skeletal system [9], [10]. In the adult, expression is nearly ubiquitous and promotes normal turnover and maintenance of ECM. As part of normal wound healing following injury, there is a marked induction of *Ctgf* expression [11]. Essentially, CTGF acts as a central coordinator of multiple pro- and anti-fibrotic signals and misregulation of CTGF is believed to result in tissue fibrosis and scarring [11], [12]. Increased *Ctgf* expression is clearly associated with fibrosis in multiple tissues including skin, liver, heart, lung, and kidney (reviewed in [11], [12]). In addition, CTGF is a diffusible protein and can be detected in urine and blood [13], [14]. A critical difference between healthy healing and pathological fibrosis may be the temporal regulation of, and/or the level and duration of, *Ctgf* expression. Thus, it is postulated that tight regulation of *Ctgf* expression is necessary to maintain healthy tissues and induce healthy, non-fibrotic healing [15].

To examine the role of CTGF *in vivo*, several animal models have been created. The first is a traditional knockout, which when homozygous, is perinatally lethal. The pups are born, but do not survive due to skeletal deformity, including a malformed rib cage which causes an inability of pups to breathe properly. The heterozygous knockouts survive and have little discernable fibrosis-related phenotype [16]. Transgenic models of *Ctgf* overproduction in bone and cartilage, cardiomyocytes, kidney podocytes, hepatocytes, lung epithelia and fibroblasts have also been made using promoter-enhancers constructs specific to each cell type [17]–[23]. The fibrotic phenotypes of these strains vary widely ranging from no increase in fibrosis to a clear fibrotic phenotype. In all of these models the relationship between gene expression level and phenotype is not clear since the normal control of *Ctgf* gene expression is disrupted and the mechanism by which altered steady state *Ctgf* gene expression influences non-target tissues has not been described. Thus, the role of *Ctgf* expression in fibrosis remains unclear and there is a need for animal models with variable *Ctgf* expression in which there is minimal alteration in the natural transcriptional regulation to clarify how altered *Ctgf* expression levels may predispose or change the progression of heart disease and other fibrotic diseases.

Here we describe an innovative method of modulating gene expression by making one animal that can express either low or high levels of a gene. This is made possible by use of Cre-recombinase to alter expression *in vivo* from low to high. The endogenous 3′-untranslated region (3′UTR) is replaced with a cassette containing the 3′UTR from the FBJ osteosarcoma oncogene (*c-Fos*, ENSMUSG00000021250) and the 3′UTR from the bovine growth hormone (*bGH*) gene, also known as somatotropin (ENSBTAG00000017220), placed in tandem. Each 3′UTR modulates the stability of the mRNA transcript and can respectively decrease (*c-Fos*) or increase (*bGH*) steady state levels of mRNA in both cells and whole animals [24]–[27].

Using this method, we have created a single construct that allows the examination of the role of *Ctgf* when it has reduced expression, increased expression, or when expression is altered in a tissue-specific manner. Our results show that *Ctgf* expression reduced to 30% of wild type is survivable, while expression nine fold higher than wild type leads to embryonic lethality by day E12.5. Thus, we have created a 30-fold range of *Ctgf* expression *in vivo*, which should prove useful in further understanding the function of CTGF in fibrotic disease.

## Results

### Global, conventional KO of the *Ctgf* gene

**Figure 1:**
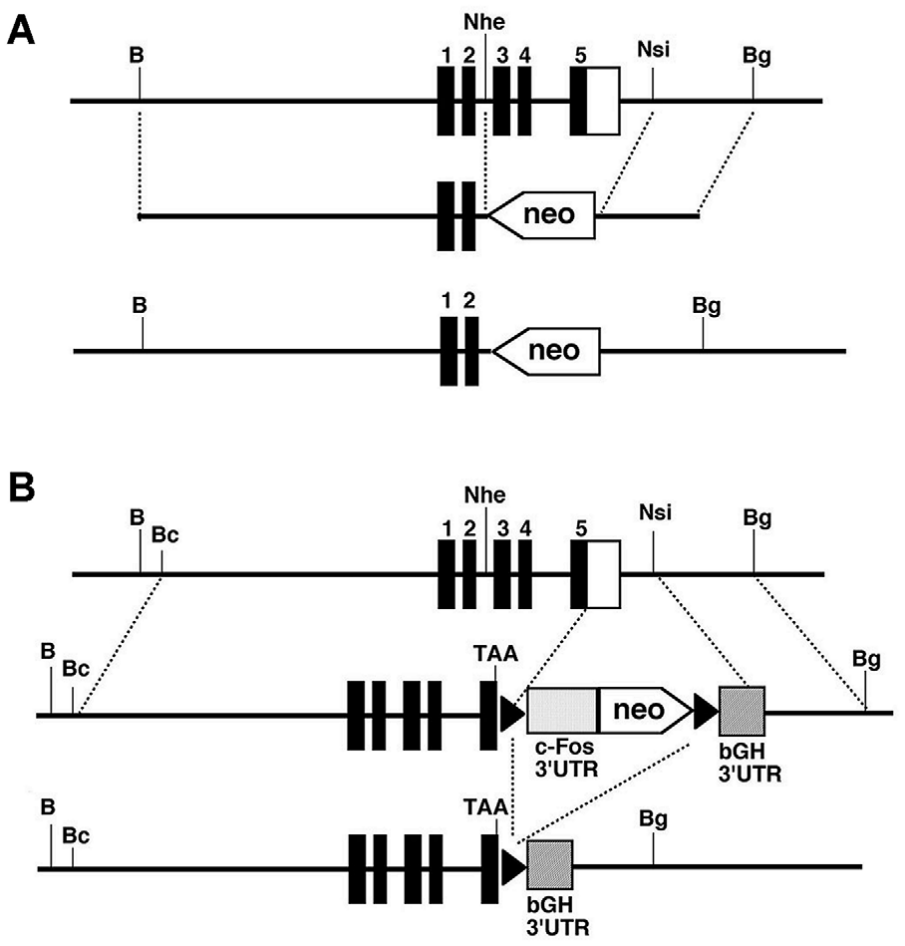
*Ctgf KO and Ctgf Lo-Hi* allele. A) *Ctgf KO* allele. Line 1 is the endogenous *Ctgf* locus. Line 2 is the *Ctgf KO* allele construct. Line 3 is the result following homologous recombination where exons 3–5 of the *Ctgf* gene were replaced with a neo cassette to generate the *Ctgf KO* allele. Black boxes indicate coding exons and a white box indicates the endogenous 3′UTR. B) *Ctgf Lo-Hi* allele. Line 1 is the endogenous *Ctgf* locus. The *Ctgf Lo-Hi* allele was generated by replacement of the endogenous *Ctgf*-3′UTR with a construct containing the *c-fos*-3′UTR and a Neo cassette flanked by loxP sites, followed by the *bGH*-3′UTR. In line 2, the modified *Ctgf* gene uses the less stable *c-Fos*-3′UTR which reduces gene expression (*Ctgf Lo* allele). In the bottom line, following Cre-mediated recombination, the *c-Fos*-3′UTR and Neo cassette are excised and the *Ctgf* gene uses the more stable *bGH*-3′UTR which increases gene expression (*Ctgf Hi* allele). TAA indicates a stop codon Restrcition sites are abbreviated B is BamH1, Bc is Bcl1, Nhe is Nhe1, Nsi is Nsi1, and Bg is Bgl2.

In order to inactivate the *Ctgf* gene, we have made a targeting construct to delete exons 3 through 5 of the *Ctgf* gene in the genome in ES cells as illustrated in Fig. 1A. The 5′ and 3′ arms of homology are a 4.7 kb BamH1/Nhe1 fragment and a 1.4 kb Nsi1/Bgl2 fragment, respectively. A neomycin resistant gene was used as a selection marker. Mice heterozygous for an inactivated *Ctgf KO* allele appear normal and express the *Ctgf* gene at approximately 50% levels. As reported by Ivkovic et al, homozygous mice completely lacking CTGF are born but die within 16 hours [16].

### Strategy to generate the *Ctgf-Lo* and *Ctgf-Hi* alleles

Our initial attempts to increase the stability of *Ctgf* transcripts in vivo using 3′UTR replacement with the *bGH*-3′UTR [24] met with difficulties. Chimeric mice with a modified allele tended to be runted and did not transmit the modified allele (data not shown). In order to circumvent the potential embryonic lethality of high *Ctgf* expression and generate adult mice overexpressing the *Ctgf* gene, we devised a novel strategy using a low-high cassette (Lo-Hi) that allows us to change gene expression levels from low to high by altering mRNA stability (Fig. 1B). The second line in Fig. 1B illustrates the *Ctgf-Lo* allele made by a replacement of the endogenous *Ctgf* gene 3′UTR sequence with a cassette that includes a loxP site, followed by the 3′UTR sequence from the FBJ osteosarcoma oncogene (*c-Fos*-3′UTR), the neomycin resistant gene (neo), a second lox P site, and the *bGH*-3′UTR. The modified allele, *Ctgf-Lo*, uses the *c-Fos*-3′UTR and so its transcripts are less stable than those from the wild type *Ctgf* allele. However, a Cre-mediated recombination between the two loxP sequences in the Lo-Hi cassette excises the *c-Fos*-3′UTR and the neo gene and generates the *Ctgf-Hi* allele that uses *bGH*-3′UTR (Figure 1B, third line). The transcript with the *bGH*-3′UTR is more stable than normal and consequently steady state levels of *Ctgf* mRNA increase.

### Mice with low *Ctgf* expression

Heterozygotes for the *Ctgf-Lo* (*Ctgf Lo/+*) allele are born and appear normal. *Ctgf Lo/+* mice were first intercrossed to determine if the *Ctgf Lo/Lo* phenotype is viable. Pups were born from this cross at a 10∶18∶5 genotypic ratio (*+/+*: *Lo/+*: *Lo/Lo*). The number of *Ctgf Lo/Lo* pups was about half the expected Mendelian ratio of 1∶2∶1 for the cross, but the Chi square test was not significant (p = 0.409, n = 33). The *Ctgf Lo/+* and *Ctgf Lo/Lo* pups are healthy, reproduce normally, and are indistinguishable from their wild type and heterozygous littermates.

**Figure 2:**
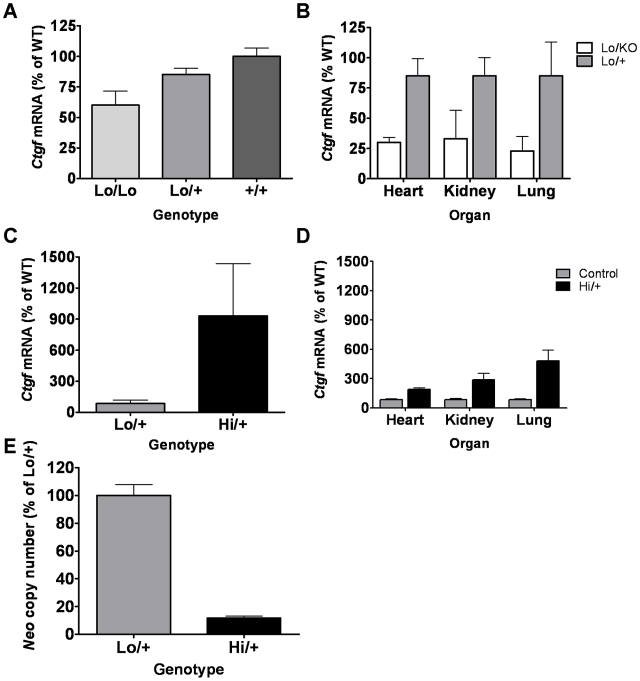
*Ctgf* mRNA levels by quantitative RT-PCR. A) *Ctgf* mRNA expression from tail samples of 10 day pups from a cross of *Ctgf Lo/+* parents. All mice are siblings. The mean of *Ctgf* +/+ (wild type) expression is set to 100% expression *LoLo*: n = 5, *Lo/+*: n = 25, and *+/+*: n = 21. B) *Ctgf* mRNA expression from tissues of five month old mice. *Ctgf Lo/+* mice are the control with *Lo/+* expression set to 85% based on results from Figure 2A. For each organ *Lo/+*: n = 14 and *Lo/KO*: n = 8. C) *Ctgf* mRNA expression from whole mouse embryos at day points E11.5, normalized to *Lo/+* the same as Figure 2B. *Lo/+*: n = 8 and *Hi/+*: n = 13 D) *Ctgf* mRNA expression from tissues of 10 month old mouse tissues normalized to a combined control of animals of the genotypes *+/+;Cre-*, *Lo/+;Cre-,* and *+/+;Cre^+^*. E) Neo gene copy number in embryos at day E11.5. Copy number is in arbitrary units and normalized to *Lo/+* = 100%. *Lo/+*: n = 18 and *Hi/+*: n = 28. For all graphs the *Lo/+* animals are heterozygous for the *Ctgf Lo* allele and do not have a Cre allele. The *Hi/+* animals are heterozygous for the *Ctgf Hi* allele and have one EIIa-Cre allele.

The *Ctgf Lo/Lo* mice are expected to have reduced *Ctgf* mRNA expression throughout the body. Tail snips from 10 day old pups were used to assay for *Ctgf* mRNA expression. *Ctgf Lo/Lo* mice express about 60% of wild type levels of *Ctgf*. The *Ctgf Lo/Lo* mice are similar in *Ctgf* expression level to the *Ctgf* heterozygous knockout (*Ctgf KO/+*) mice which express 50% of wild type *Ctgf*. *Ctgf Lo/+* heterozygotes have an intermediate *Ctgf* expression level that is 85% of wild type mice (*+/+*: 100%±6.8, n = 21, *Lo/+*: 85%±5.1, n = 25, p<0.05, *Lo/Lo*: 60%±11.4, n = 5, p<0.005, Figure 2A).

### Mice with very low *Ctgf* expression

*Ctgf Lo/Lo* mice were crossed to *Ctgf* heterozygous knockouts (*Ctgf KO/+*) to generate mice with very low *Ctgf* expression (*Ctgf Lo/KO*). *Ctgf Lo/KO* mice were born at the expected Mendelian ratio of 1∶1 (17 *Lo/+*: 15 *Lo/KO* **)**. Tissues from adult siblings (males and females) were assayed for *Ctgf* mRNA expression by RT-PCR. *Ctgf Lo/KO* heart tissue had significantly lower *Ctgf* expression (heart: *Lo/KO*: 30%±4.2, n = 7, *Lo/+*: 85%±14.3 for, n = 14, p = 0.002, Figure 2B). Similarly, both kidney and lung had significantly reduced *Ctgf* expression (kidney: *Lo/KO*: 33%±23.5, n = 8, *Lo/+*: 85%±15.0, n = 14, p = 0.01 and lung: *Lo/KO*: 23%±11.9, n = 8, *Lo/+*: 85%±27.9, n = 14, p = 0.02, Figure 2B). When the results from various tissues are combined, *Ctgf* expression in *Lo/KO* mice is on average 29% of wild type ((30%+33%+23%)/3 = 28.7%). The *Ctgf Lo/KO* animals reproduce adequately, are healthy, phenotypically normal in appearance, and display no overt structural phenotypes.

### Embryonic lethality in mice with high *Ctgf* expression on a B6.129 mixed background

When Cre is present, a Cre-mediated recombination between the two loxP sequences within the *Ctgf-Lo* allele excises the *c-Fos*-3′UTR and the neo gene, this generates the *Ctgf-Hi* allele that uses the *bGH*-3′UTR. To generate mice carrying a *Ctgf-Hi* allele, *Ctgf Lo/+* mice on a B6.129 mixed genetic background were crossed to a heterozygous whole body Cre expressing mouse line 129.Tg(Ella-Cre) (from B6.FVB-Tg(EIIa-cre) C5379Lmgd/J stock that was backcrossed to 129/SvEv for 18 or more generations). Heterozygotes for the *Ctgf Hi* allele (*Ctgf Hi/+* mice) were defined as presence of the *bGH*-3′UTR, presence of a Cre allele and low Neo gene copy number. After seven litters and 40 pups, no live mice carrying both the modified *Ctgf* allele and the EIIa-Cre transgene were recovered, suggesting the *Ctgf-Hi* allele causes an embryonic lethal phenotype.

To examine the timing and nature of the lethality, we used timed matings and recovered embryos between E13.5 and E14.5. At this embryonic age, *Ctgf Hi/+* embryos were recovered at a ratio of 10∶15∶11∶7 (*+/+;Cre-*: *Lo/+;Cre-*: +/+;*Cre^+^*: *Hi/+;Cre^+^*) which was not different from the expected Mendelian genotypic ratio of 1∶1∶1∶1 (Chi Square, p = 0.385, n = 43).

**Figure 3:**
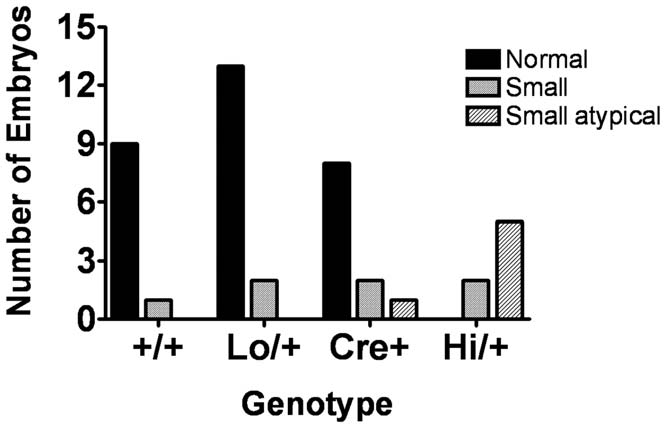
Phenotype of E13.5 embryos. Embryos are from a cross between *Ctgf Lo/+* and *EIIa-Cre +/-* parents. The number of embryos for each genotype classified as Normal, Small, or Small atypical. *+/+* n = 9, *Lo/+* n = 15, *Cre^+^* n = 11, *Hi/+* n = 7. The +/+ animals are wild type for *Ctgf* and do not have a Cre allele, *Lo/+* animals are heterozygous for the *Ctgf Lo* allele and do not have a Cre allele. *Cre^+^* are wild type for *Ctgf* and have one Cre allele. And *Hi/+* are heterozygous for the *Ctgf Hi* allele and have one Cre allele.

**Figure 4:**
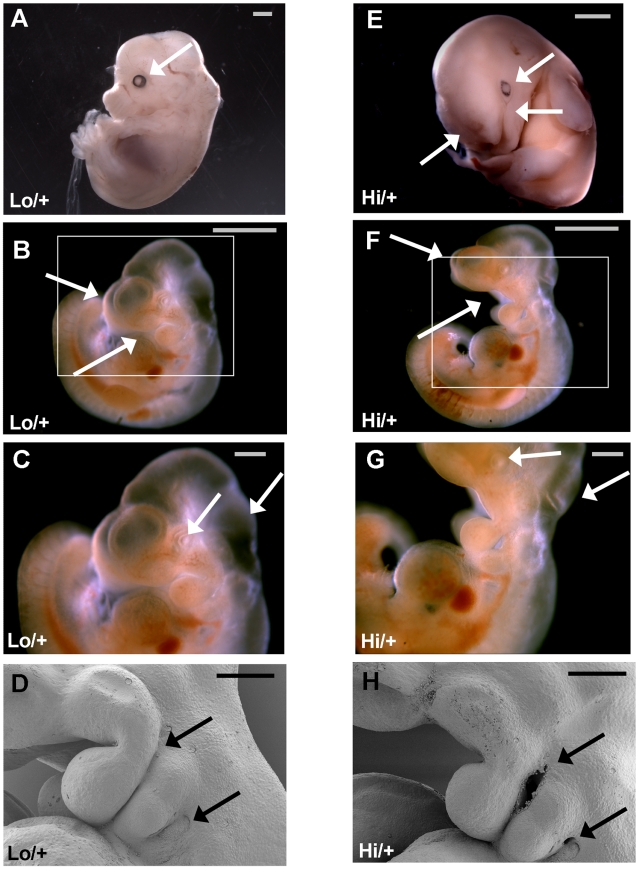
E14.5 and E10.5 embryos. A) and E) are fixed embryos at E14.5 from a cross between *Ctgf Lo/+* and *EIIa-Cre +/−* B6.129 mixed background parents. A) is a normal *Lo/+* and E) is a small atypical *Hi/+* at higher magnification. Top arrow denotes less developed eye in E) and the lower two arrows point to lateral and midline facial clefting, respectively. Embryos B) to D) and F) to H) are E10.5 embryos from a cross between *Ctgf Lo/Lo* and *EIIa-Cre +/−* 129/SvEv background parents. B), C), and F), G), are unfixed embryos. B), C) and D) are normal *Ctgf Lo/+* embryos. F), G), and H) are *Ctgf Hi/+* embryos. The top arrow in B) and F) points to the forebrain which appears smaller in F) and the lower arrow points to the mouth gape in F) also indicative of a small forebrain. The top arrow in C) and G) points to the eye and the bottom arrow points to the hindbrain which both appears abnormal in G). The pictures in D) and H) are from scanning electron microscopy. The arrows in D) and H) point to the first (top) and second (bottom) pharangeal (branchial) clefts. In D) there is normal closure of the clefts and in H) the clefts are abnormally patent (open). B) and C) are the same embryo and F) to H) are the same embryo. The bars in A), B), E), and F) represent 1000 µm and in C), D), G), and H) the bars represent 250 µm.

At recovery, embryos were weighed and phenotypically classified as normal, small, or small atyical without knowledge of the genotype. Normal embryos were nondysmorphic in appearance, of the expected developmental stage for the embryonic age, and were within two standard deviations of the mean embryo weight for that litter. Small embryos were nondysmorphic in appearance and of the expected developmental stage for the embryonic age, but small in size with a body weight below two standard deviations of the litter average (litter averages include all pups that were sufficiently intact to be weighed). Small atypical embryos were developmentally delayed, macerated, or poorly vascularized with a body weight below two standard deviations of the litter average. By this classification method, no normal *Ctgf Hi/+* embryos were present among 43 embryos at E13.5-E14.5 (Figure 3). All of the *Ctgf Hi/+* embryos were either small or small atypical and were similar in appearance to the examples in Figure 4E, which would be classified as small atypical (Fig. 4A is a normal embryo from the same litter). In addition to small size and weight, the *Ctgf Hi/+* embryos generally lacked clearly visible vasculature, lagged behind in development, and many were macerated and likely to be in the process of reabsorption (Figure 4E). This suggests that the embryos begin to die before E13.5. While the reason for the lethality is unclear, we observed a less developed eye (4e top arrow), as a mark of developmental delay and craniofacial defects including both lateral and midline facial clefting (4e middle and bottom arrow, respectively). No *Ctgf Hi/+* embryos were recovered from the litters older than embryonic day point 14.5.

### Embryonic lethality in mice with high *Ctgf* expression on a 129/SvEv background

In order to investigate the timing of the lethality more closely and to examine embryo viability in a pure 129/SvEv background, *Ctgf Lo/Lo* mice on a 129/SvEv background were crossed with 129.Tg(Ella-Cre) mice. After genotyping three litters, no *Ctgf Hi/+* embryos were recovered at E13.5, suggesting an earlier lethality in the fully inbred background. Embryos on a 129/SvEv background were then harvested at E10.5 (Figure 4 B-D and F-H) for examination of morphology.

The *Ctgf Hi/+* embryos at E10.5 display developmental delay and a number of morphological defects. The gapping open mouth posture seen in Figure 4F (bottom arrow) and 4G is suggestive of a small forebrain (also 4B and 4F top arrow) and the abnormal indentation of the back of the head seen in Figure 4G (bottom arrow) suggests an abnormal hindbrain. The embryo in Figure 4G also appears to have less visible microvasculature in the face and possibly elsewhere and has a less developed eye (top arrow 4 g) suggesting possible developmental delay. Additionally, the closure of the pharyngeal (branchial) arches, particularly between the first and second (pharyngeal cleft one) and second and third arch (pharyngeal cleft two), were abnormally patent (compare Figure 4D to 4H).

No *Ctgf Hi/+* embryos on a 129/SvEv inbred background were recovered beyond E12.5 suggesting the lethality on the 129/SvEv background is earlier than that of the B6.129 background, at around day E11.5-E12. Despite the earlier time point of lethality in the 129/SvEv background, developmental delay and craniofacial defects were present in both backgrounds.

### Gene expression levels of *Ctgf Hi/+* embryos

In order to examine the expression levels of the *Ctgf-Hi* allele, *Ctgf Lo/Lo* females were crossed to 129.Tg(Ella-Cre) Cre^+^ males and the embryos were harvested at E11.5 and assayed for whole body *Ctgf* gene expression using RT-PCR. *Ctgf* expression varied greatly in *Ctgf Hi/+* embryos, ranging between two and fifty times greater than the mean of *Ctgf Lo/+* embryos. Most likely this is due to variations in developmental stage, embryo condition, and variations in Cre expression. On average the *Ctgf Hi/+* embryos had a greater than ten fold increase in *Ctgf* mRNA levels compared to the *Ctgf Lo/+* littermate controls, which is a nine fold increase in *Ctgf* expression over WT (*Lo/+*: 85%±34, n = 8 and *Hi/+*: 930%±506, n = 13, p<0.05, Figure 2C).

To validate that excision of the *c-fos*-3′UTR and neo gene was occurring, whole embryos were assayed for neo gene copy number as a measure of excision efficiency. On average *Ctgf Hi/+* embryos had less than one eighth the amount of neo alleles than the *Ctgf Lo/+* embryos (*Lo/+*: 100%±7.8, n = 18 and *Hi/+*: 11.6%±1.7, n = 28, p<1×10^−7^, Figure 2E). While the excision of the *c-fos*-3′UTR and neo is not complete, the switch from the *Lo* allele to the *Hi* allele is occurring efficiently and in the large majority of cells. There did not appear to be a direct relationship between reduction in neo copy number and increase in *Ctgf* gene expression levels suggesting that mechanisms other than just efficiency of *c-fos*-3′UTR and neo excision were regulating *Ctgf* gene expression levels in the embryos (data not shown).

### Pattern of CTGF protein in embryos

**Figure 5:**
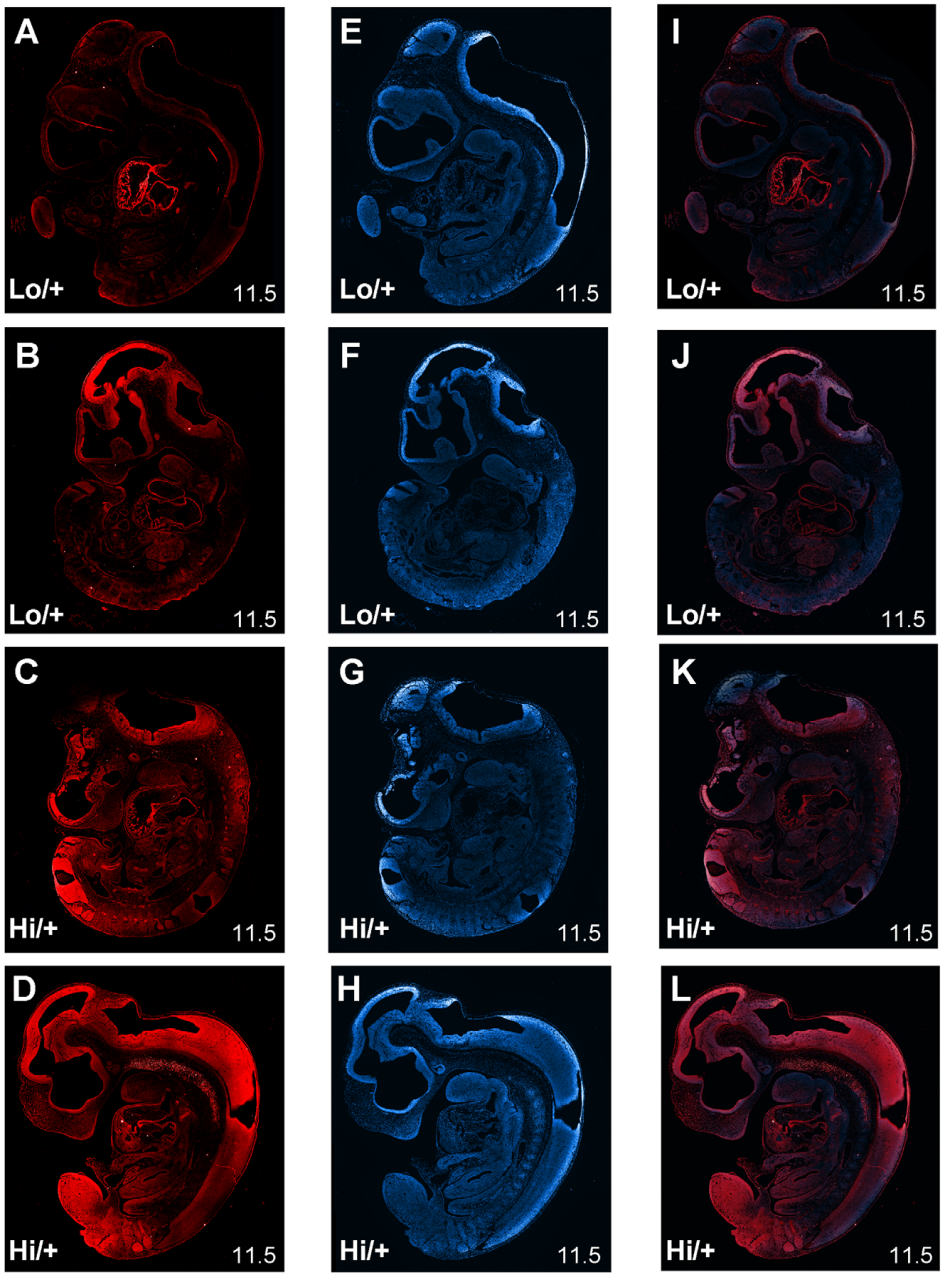
Immunofluorescence of E11.5 *Ctgf Lo* and *Hi* embryos. Panels A) – D) are stained with an anti-CTGF antibody and a Alexa 594 (Texas Red) secondary. Panels E) – H) are stained with DAPI to highlight nuclei. Panels I) – L) are a merge of the Red and the DAPI layer. Genotypes for each panel are as marked on the panel. The panels in each row are from the same section. All panels are at 5× magnification.

Increased *Ctgf* mRNA levels may be causing changes in the levels or pattern of CTGF protein in *Ctgf Hi/+* embryos. Thin sections of embryos were stained with a CTGF antibody and a fluorescent seconday then counterstained with DAPI to visualize the level and pattern of CTGF protein. CTGF was visible in almost evey tissue of the embryos and appeared to be primarily staining the extracellular matrix and the cell surface (Fig. 5I–5L). The *Hi/+* embryo in Fig. 5D has the highest CTGF staining and the *Lo/+* embryo in Fig. 5A the lowest staining, suggesting a genotype dependent difference CTGF levels. Although the heart in Fig. 5A is staining strongly, this likely represents a specific and perhaps short-lived induction of CTGF in the heart during embryogenesis. The other two embryos (Fig. 5B and 5C) have similar staining intensity despite being different genotypes suggesting CTGF levels vary substantially within each genotype. For the most part individual differences in staining were greater than the differences between genotypes, and there was not a distinct difference in the pattern of CTGF staining nor a dramatic difference in the overall intensity of CTGF staining between *Ctgf Lo/+* and *Ctgf Hi/+* (Figure 5). Immunohistochemistry (IHC) with a higher antibody concentration and E13.5 mixed genetic background embryos yielded a similar result (data not shown).

### Survivors with high *Ctgf* gene expression

**Figure 6:**
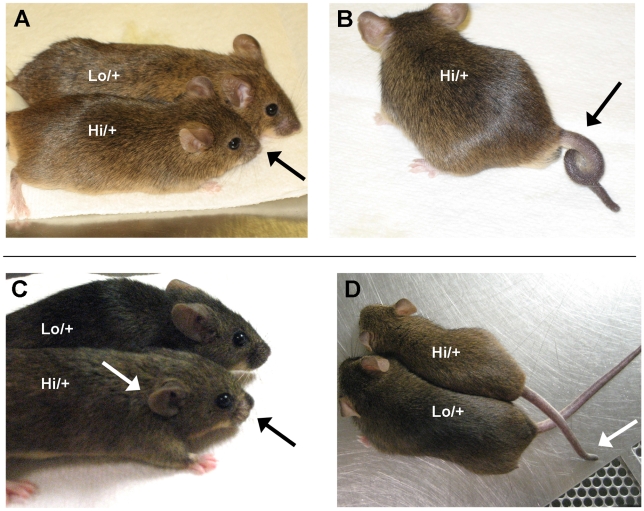
Survivors of *Ctgf Hi/+* embryonic lethality. Gross morphology of high *Ctgf*-expressing survivors at 10 months old. Males are shown in A) and B) and females are shown in C) and D). In A) and B) the *Ctgf Hi/+* mice (foreground) have small ears, shortened face and short body length. B) The short curly tail of the *Hi/+* male. D) Short kinked tail and short body of the *Hi/+* female (top) compared with a control sibling (bottom). All the animals shown are siblings from a cross between *Ctgf Lo/+* and *EIIa-Cre +/−* animals on a mixed B6.129 background. The controls (background of A) and C) and bottom of D)) are of the genotype *Ctgf Lo/+*.

After many matings, four *Ctgf Hi/+* animals (out of 81 that were genotyped) escaped embryonic lethality and survived to adulthood (26∶28∶23∶4 ratio of *+/+,Cre−*: *Lo/+,Cre−*: *+/+,Cre^+^*: *Hi/+,Cre^+^*). One male and one female of the surviving animals were siblings from a mixed B6.129 mating. Two additional male survivors came one year later, one from a mixed B6.129 mating and one from a 129/SvEv mating. All four mice exhibited the same phenotype: a shortened face, small ears, a shortened and kinked or curled tail, and a shortened overall body length (Figure 6).

The surviving *Ctgf Hi/+* animals and their sibling controls were further characterized by dual energy x-ray absorptiometry (DEXA) to assess whole body: bone mineral density (BMD), bone mineral content (BMC) and body composition (lean, fat and % fat mass). Although the number of samples was small and the animals were in different genetic backgrounds, all of the *Ctgf Hi/+* animals had significantly smaller bone area and lean mass than controls (bone area: controls: 9.87±0.33 cm^2^ and survivors: 8.35±0.57 cm^2^; lean mass: controls: 25.4±1.1 grams and survivors: 21.0±0.95 grams, controls: n = 9 and survivors: n = 4, p<0.05, Table 1) indicating a smaller overall body size as suggested by their outward appearance. The BMC of *Ctgf Hi/+* survivors was low suggesting changes in the bone mineralization or morphology (controls: 0.642±0.033 grams, n = 9 and survivors: 0.532±0.052 grams, n = 4, p<0.05, Table 1).

**Table 1:** DEXA and necropsy measurements of *Ctgf Hi/+* survivors and sibling controls.

	Measurement	Control	Hi/+; Cre +	T-Test p-value
**Bone (DEXA)**	BMD (g/cm^2^)	0.0648	0.0635	0.357
	BMC (grams)	0.642	0.532	**0.048**
	Area (cm^2^)	9.87	8.35	**0.016**
**Tissue (DEXA)**	Lean (grams)	25.4	21.0	**0.013**
	Fat (grams)	11.3	9.9	0.312
	Total (grams)	36.7	30.8	**0.072**
	% Fat	30.0	30.0	0.485
**Weight (Fresh)**	Total	35.720	29.057	**0.037**
	Kidney	0.221	0.170	**0.007**
	Heart	0.167	0.142	0.081
	Lung	0.133	0.097	**0.017**
**% of Body Weight**	Heart/Total	0.475	0.485	0.347
	Kidney/Total	0.311	0.300	0.329
	Lung/Total	0.381	0.343	0.268

The top half of table is DEXA measurements of live mice at 10 months of age and the bottom is necropsy data. Mice were euthanized at 10 months of age and total body weight and organ weights were measured. Males and females were pooled. The p-value for each measurement is from Student T-test. Statistically significant p-values are shown in black. Controls: n = 9 and *Hi/+*: n = 4. Genotype of controls: *Lo/+*: n = 4, *Cre^+^*: n = 3, and *+/+*: n = 2. *Cre^+^* animals are wild type for *Ctgf* and *+/+* animals are wild type for *Ctgf* and do not have a Cre allele. BMD  =  bone mineral density and BMC  =  bone mineral content.

At sacrifice several tissues including fat pads were collected and weighed to determine any gross differences. There were no differences in total amount or relative amounts of renal, inguinal, or mesenteric fat (data not shown). Organ weights tended to be smaller with both kidney and lung significantly smaller in the *Ctgf Hi/+* survivors (Kidney: controls: 0.221±0.010 grams and survivors: 0.170±0.013 grams; Lung: controls: 0.133±0.009 grams and survivors: 0.097±0.008 grams, controls n = 9 and survivors n = 4, p<0.05, Table 1). However, organ weights as a ratio of total body weight tended to be smaller in *Ctgf Hi/+* survivors but were not significantly different from the controls (Table 1). The organ weight ratios were not different from controls because at sacrifice the *Ctgf Hi/+* survivors were smaller in total body weight than controls (controls: 35.720±1.65 grams, n = 9 and survivors: 29.057±3.57 grams, n = 4, p<0.05, Table 1) suggesting that *Ctgf Hi/+* survivors have a normally proportioned but smaller body size.

Heart, kidney and lungs from *Ctgf Hi/+* survivors and siblings or age-matched controls were harvested and assayed for *Ctgf* gene expression using RT-PCR. There was a significant increase in *Ctgf* expression in each tissue (Figure 2D). The heart had a modest increase at two-fold above controls, kidney is three-fold increased, and lung had the largest change at nearly five-fold increased *Ctgf* expression over sibling controls (Heart: *Lo/+*: 85%±9.6, *Hi/+*: 190%±16.6, p<0.0001; Kidney: *Lo/+*: 85%±11.6, *Hi/+*: 286%±67.9, p<0.001; Lung: *Lo/+*: 85%±10.1, *Hi/+*: 482%±111.6, p<0.0001, n = 9 controls and n = 4 survivors, Figure 2D). At the time of sacrifice, 10 months of age, the *Ctgf Hi/+* survivors appeared healthy and did not seem to have accelerated aging. Histological evaluation of sections of heart, kidney, and lung was not remarkable and not different from age matched controls (data not shown).

### Global increase of *Ctgf* gene expression in adult mice

**Figure 7:**
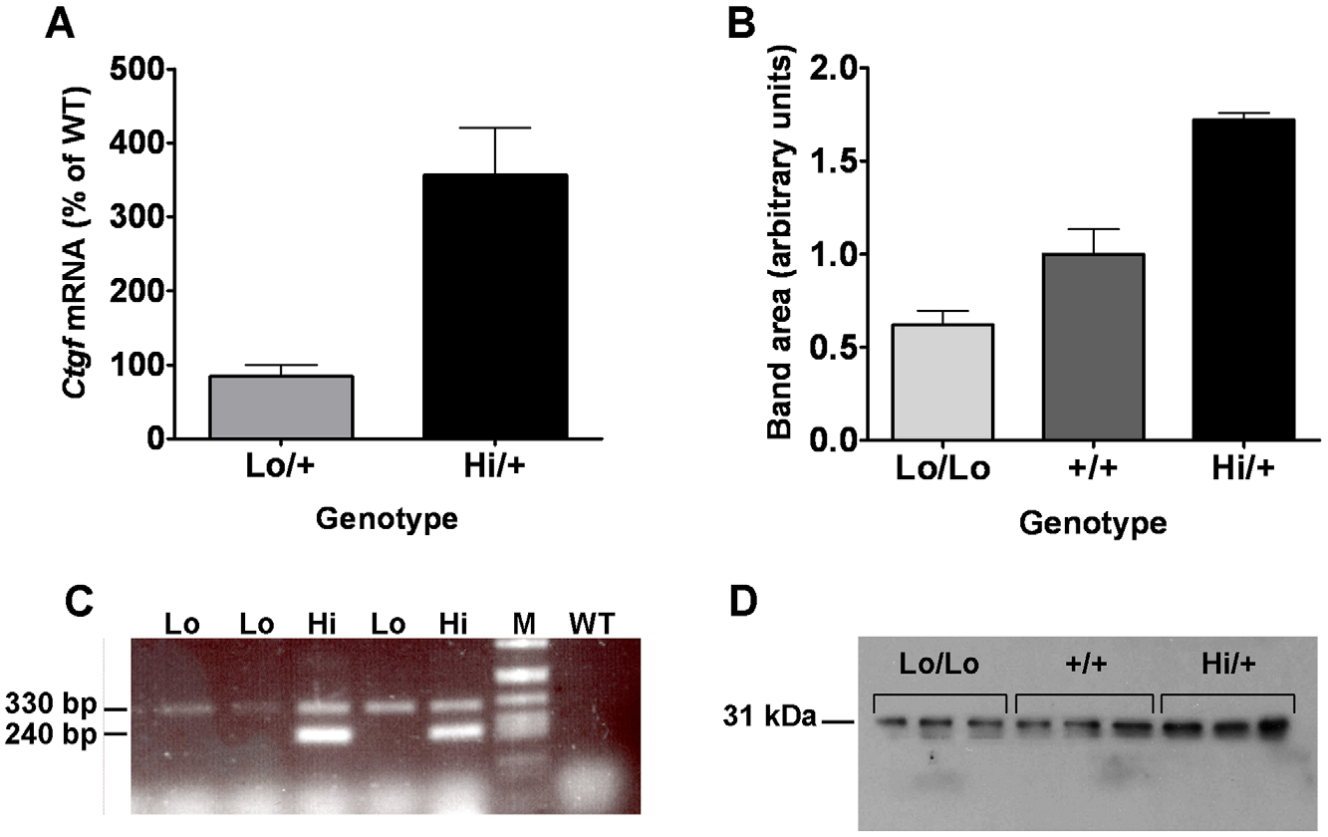
*Ctgf* mRNA levels in tamoxifen treated adults and CTGF protein abundance in plasma. A) *Ctgf* mRNA determined by RT-PCR in tamoxifen treated mice from a cross of *Ctgf Lo/Lo* and *CAG-Cre +/−* animals on a B6.129 F1 background. All animals were treated with tamoxifen and *Lo/+* is set to 85% as a control. *Lo/+*: n = 23 and *Hi/+*: n = 20. B) Quantification of CTGF protein abundance in plasma as calculated using ImageJ. *Lo/Lo*: n = 5, *+/+*: n = 5, and *Hi/+*: n = 7 animals for each genotype. C) A 330 bp band corresponds to the *Ctgf Lo* allele and a 240 bp band corresponds to the *Ctgf Hi* allele. The PCR reaction is not quantitative but the presence of the 240 bp band indicates that Cre-mediated excision of DNA flanked by the loxP sequences has taken place. Presence of 330 bp band in animals with *Ctgf Hi* allele (Hi) indicates that the excision is not complete. DNA from the wild type animal (WT) does not amplify with these primers indicating the absence of the *Ctgf Lo* allele. The lane of size markers is indicated by M. D) A representative western showing a doublet band for CTGF just above 31 kDa for three *Ctgf Lo/Lo* mice, three WT mice and three *Ctgf Hi/+* mice. In A) *Lo/+* animals are heterozygous for the *Ctgf Lo* allele and do not have a Cre allele. In A), B), and D) *Hi/+* animals are heterozygous for the *Ctgf Hi* allele, have one tamoxifen-inducible CAG-Cre. All animals have been treated with tamoxifen.

The survival of several animals with very high *Ctgf* expression suggests that while high *Ctgf* expression is embryonic lethal, in an adult it is compatible with survival. To test this hypothesis we crossed *Ctgf Lo/Lo* mice with CAG-Cre mice, which are a tamoxifen-inducible global Cre expresser strain [28]. At four to six weeks of age, offspring were treated with tamoxifen to induce Cre expression. PCR of small skin biopsy of tamoxifen-treated animals showed that the *Lo-Hi* allele functioned as predicted with excision of the *c-fos*-3-UTR and neo (*Lo* allele) to generate the smaller the *Hi* allele in the presence of tamoxifen and the inducible Cre gene (Figure 7C). Similar to *Hi/+* survivors and embryos, *Ctgf* gene expression in Cre expressing mice (*Ctgf Hi/+* mice, after tamoxifen treatment) was approximately four-fold higher than in *Ctgf Lo/+ mice* without the Cre gene (*Lo/+*: 85%±15.3, n = 23 and *Hi/+*: 357%±63.4, n = 20, p>0.00005, Figure 7A).

Three months after tamoxifen treatment both males and females remained indistinguishable from the non-Cre carrying littermates, suggesting that high levels of *Ctgf* expression in the adult are survivable. This experiment also shows that our construct can be used in an inducible fashion to study fibrotic disease in adult animals.

### CTGF protein levels in plasma

To confirm that changes in *Ctgf* mRNA expression translated to changes in CTGF protein abundance, a Western blot of plasma samples with a mouse-specific CTGF antibody was performed for quantitation. CTGF is a secreted protein and is known to circulate in plasma at measurable levels. Recent human and mouse research has suggested that CTGF protein levels in the plasma can be used as a non-invasive marker of *Ctgf* gene expression levels [29], [30]. Band sizes ranging from 11 kDa to the full length 38 kDa have been reported in Western blots for CTGF, including multiple reports of a doublet around 31 kDa [31], [32]. The primary band we observed for CTGF in adult plasma was a doublet around 31 kDa (Fig. 7D). Quantitation by densitometry showed that the *Ctgf Lo/Lo* mice had significantly reduced CTGF protein to a level that was about 60% of wild type (*Lo/Lo*: 0.62±0.077, n = 5, *+/+*: 1.00±0.135, n = 5, p<0.05, Figure 7B), which is similar to the *Ctgf Lo/Lo* mRNA expression levels (Figure 2A). In contrast, tamoxifen-treated *Ctgf Hi/+* animals had significantly increased CTGF protein abundance of about 170% of wild type (*Hi/+*: 1.72±0.037, n = 7, p<0.0001, Figure 7B). While this is not as high as the mRNA expression levels it is a significant and very consistent increase in CTGF protein levels.

## Discussion

Previous studies have shown that altering or interchanging the 3′UTR of a gene is an effective method for altering gene expression in cells and in animals [24]–[27]. The method takes advantage of the idea that the 3′UTR, in part, regulates mRNA abundance primarily through modulating mRNA stability. In altering the stability of a gene's mRNA, the mRNA abundance and thus the protein abundance can be modulated in a qualitatively predictable manner. By combining Cre-lox technology with the use of two well-characterized 3′UTR sequences, *c-Fos*-3′UTR and *bGH*-3′UTR, a single allele that can decrease or increase gene expression has been assembled. This method was applied to the gene *Ctgf* to generate an allelic series that has a 30-fold range of *Ctgf* expression as summarized in Table 2. In addition, in line with previous reports by Ivkovic et al [16], our *Ctgf KO/KO* (homozygous knockout) are born but die within a day.

**Table 2:** Strains in the *Ctgf* allelic series.

*Ctgf* genotype	Allele	*Ctgf* mRNA Expression
KO/KO	del/del	0%
Lo/KO	*c-fos*-3′/del	30%
+/KO	WT/del	50%
Lo/Lo	*c-fos*-3′/*c-fos*-3′	60%
Lo/+	*c-fos*-3′/WT	85%
+/+ (WT)	WT/WT	100%
Hi/+ inducible	*bGH*-3′/WT	360%
Hi/+ embryos	*bGH*-3′/WT	930%

*Ctgf* expression levels are relative to WT. A single WT allele expresses 50% (2×50% = 100%). Each copy of the *Ctgf Lo* allele expresses about 30% of WT (*Lo/+* = 30%+50%≈85%). Each copy of the *Ctgf Hi* allele expresses about 300% to 860% of WT. Abbreviations: KO is knockout, del is exon 3–5 deletion, and 3′ is 3′UTR.

We found that embryos with a *Ctgf Hi* allele had increased *Ctgf* mRNA levels that were nine-fold higher than WT in embryos and the increase in *Ctgf* expression was embryonic lethal, depending on genetic background, between day E11 and E13.5. The lethality of the *Ctgf Hi* allele, made from the *Lo-Hi* allele, recapitulates the lethality observed in our previous attempts to directly generate a *Ctgf Hi* allele with the *bGH*-3′UTR. Embryos from both 129/SvEv and B6.129 mixed backgrounds display craniofacial defects consistent with alterations to the first and second pharyngeal arches. The fissures between the arches in the mutant E10.5 embryo in Figure 4H do not have a jagged border or other morphology that indicates a handling-related tear. In addition, similar fissures were observed in two other *Ctgf Hi/+* embryos, suggesting that this defect was a result of high *Ctgf* gene expression. Defect of the pharyngeal arches, rostrum and midline in *Ctgf Hi/+* embryos are in congruence with previous reports that *Ctgf* is highly expressed in these regions at E9.5-E10.5 [16]. In humans, altered development of the first and second pharyngeal arches is known to cause malformations of the ear, cheekbone, upper and lower jaw, soft palate, eye, and facial muscles and nerves [33]. In particular, closure defects of the first and second pharyngeal clefts (the spaces between the arches) are associated with oblique or lateral clefting and cervical/branchial (neck) fistula [34].

The generalized developmental delay and craniofacial defects like those seen in *Ctgf Hi/+* embryos by themselves are not necessarily lethal [35], so it remains unclear why the *Ctgf Hi* allele is embryonic lethal. Craniofacial defects are often observed in conjuction with cardiac defects and both phenotypes are thought to be caused by improper migration and differentiation of neural crest cells [36], [37]. Defects including craniofacial dysmorphism and abnormal pharyngeal arches as well as cardiac defects were observed in transforming growth factor (*TGFβ*) family knockout mutant mouse strains and were thought to be caused by defective neural crest cell migration [36]. Although, *TGFβ* isoforms are most commonly thought to increase *Ctgf* expression [38], there are recent reports that suggest *Tgfβ2* can inhibit *Ctgf* expression [39]. If TGFβ2 is regulating CTGF *in utero*, loss of *Tgfβ2* could lead to increased levels of CTGF, creating a similar phenotype to our model of increased *Ctgf* expression. Therefore, dysfunction of neural crest cells, which likely causes the craniofacial defects in *Ctgf Hi/+* embryos, may be the ultimate reason for the embryonic lethality. No overt defects were observed in the heart in our limited histological analysis, and immunohistochemical staining revealed that *Ctgf* expression may be generally increased in *Hi/+* embryos but is not consistent. Despite the large increase in mRNA, immunohistochemistry (IHC) does not show large differences in CTGF levels suggesting that IHC may not be sufficiently sensitive or that translational regulation may be occurring. Future experiments using *in situ* hybridization and/or tissue-specific overexpression of *Ctgf* should focus on defects in neural crest cells, especially in the heart, as a possible cause for the *Ctgf Hi/+* embryonic lethality.

Alternative causes for lethality in *Ctgf Hi/+* embryos include failures of vasculogeneis or erythropoeisis, both are crucial developmental milestones of E10-E12 embryos [40]. Many of the *Ctgf Hi/+* embryos recovered appeared generally pale and may have lacked proper circulation. Vascular endothelial growth factor (VEGF) is known to be an essential factor in vasculogenesis and is thought to have a complex regulatory relationship with CTGF [41]–[43]. Disruption of VEGF signaling caused by high CTGF levels could be the cause of this defect. Failures in placental development, cell migration, or hematopoietic proliferation at this embryonic stage are also common reasons for embryonic lethality and could be the cause of lethality in the *Ctgf Hi/+* embryos [44]–[46].

An unexpected benefit of the uneven penetrance of the EIIa-Cre has been the few animals that have escaped the embryonic lethality. Genotyping showed that the survivors do not have complete neo excision in tail DNA suggesting they are mosaics for the *Ctgf Lo* and *Ctgf Hi* alleles and likely escaped lethality due to low Cre expression during embryogenesis. Like the embryos, the survivors express significantly increased *Ctgf* as well as exhibit craniofacial defects in the form of a shortened rostrum. In addition, similar to previously reported *Ctgf* high expression transgenics, [17], [18] the survivors have skeletal defects in the form of shorted body-length, shortened or kinked tails, and reduced bone mineral content. All of these similarities as well as mRNA expression levels suggest that the *Ctgf Hi/+* survivors are true high expressers.

The EIIa-Cre line is known to have a mosaic pattern of Cre expression with varying excision efficiency in different tissues as well as large variability between animals in the amount of Cre-mediated excision. Previous reports have shown that with the Ella-Cre as few as 50% of animals undergo high levels of Cre-mediated excision in the first generation [47], [48]. The EIIa-Cre expression profile reported by The Jackson Laboratory shows that certain organs such as lung and kidney have very high Cre expression in adults and other organs such as heart have much lower Cre expression [49]. It is noteworthy that the pattern of gene expression observed in our survivor mice is similar to the report of Cre expression by The Jackson Laboratory and shows that *Ctgf* expression is moderately increased in heart (two-fold) and substantially increased in the kidney and lung (three-fold and five-fold, respectively).

These surviving animals are rare with only four animals surviving the embryonic lethality in two years of breeding. Matings of survivor males never produced offspring that carried a fully floxed (neo negative) high expressing allele, reinforcing that the *Ctgf Hi/+* allele is embryonic lethal. Therefore, due to their rarity and lack of direct transmission of the *Ctgf Hi/+* allele, these animals are not a tenable model for *Ctgf* overexpression.

We then used a mouse strain that expresses tamoxifen-inducible Cre gene and showed that high *Ctgf* expression in an adult is not lethal. With only a moderate dose of tamoxifen, these animals express a four-fold increase in *Ctgf* mRNA and a nearly two fold increase in CTGF protein abundance in plasma and do no appear to suffer any immediate ill effects. Unlike the low *Ctgf* expression models where decreases in protein levels paralleled that of mRNA, in the *Ctgf Hi/+* there is a two-fold difference between the protein abundance in plasma (1.7-fold increased) and the tissue mRNA gene expression (3.6-fold increased). The discrepancy may be due to the inaccuracy of the method used to quatitate the protein or to unknown regulation at the translational level. The increase in *Ctgf* mRNA was not as high as in the embryos so a higher tamoxifen dose, or treatment at a younger age, may increase *Ctgf* expression to a level closer to the nine-fold increase we observe in embryos.

Previous reports have implicated CTGF as a profibrotic signaling molecule leading to the suggestion that reducing *Ctgf* expression by treatment with a drug or a monoclonal antibody may be a worthwhile therapeutic strategy in treating fibrotic disease [11], [29], [30]. This therapeutic strategy, however, remains largely untested. The lack of overt fibrotic phenotype in histological analysis of the *Ctgf Hi/+* survivors and the externally normal phenotype of the tamoxifen treated *Ctgf Hi/+* animals suggests that genetically increasing *Ctgf* expression by itself is not sufficient to cause fibrosis. This is consistent with a report which showed that exogenous application of CTGF to the skin alone is not sufficient to cause fibrosis [50]. Transgenic mice overexpressing *Ctgf* in cardiomyocytes [19], kidney podocytes [20], and hepatocytes [21] show no spontaneous fibrosis of tissue while transgenic mice overexpressing *Ctgf* in lung respiratory epithelial cells [22], and fibroblasts [23] show abnormal thickening of the alveolar septa or dermis and fibrotic alterations [22], [23]. However, fibrosis in the lung-specific model is a largely development-related phenotype [22] and in the fibroblast-specific model the *in vivo* level of *Ctfg* expression was not established [23], so these results may differ from ours because of differences in levels of *Ctgf* expression or that the transgene is not responsive to normal transcriptional regulation of the *Ctgf* allele at the endogenous locus. Altogether, the current literature does not preclude a role for CTGF in fibrotic disease, but it suggests that activation of other factors or the application of stress may be required in addition to *Ctgf* overexpression in order to cause fibrosis.

Increased *Ctgf* expression has been observed in conjunction with fibrotic pathology in a number of organs and disease conditions. However, an unequivocal role for *Ctgf* as a causal factor in pathological fibrosis remains questionable. An allelic series with a wide range of genetically altered *Ctgf* gene expression provides a tool to test, the effectiveness of reducing *Ctgf* expression, *in vivo*, to prevent fibrotic disease and determining if increased expression of *Ctgf* is causal in fibrotic disease. In addition, by generating mouse lines with varying *Ctgf* expression using the endogenous locus, the function of *Ctgf* in fibrotic disease can be tested more effectively.

We note that this method of altering gene expression, through altering a gene's 3′UTR, avoids many of the issues of random insertion transgenes including insertional mutagenesis. Additionally, a single founder with the *Lo-Hi* allele in combination with existing resources allows the production of an allelic series of quantitative variants. We also note that, the construct can be arranged to produce a *Hi-Lo* allele which high expression allele can be switched to low expression allele. Altogether, this method will likely be particularly useful for genes with multiple separate functions, genes expressed in many tissues, as well as in cases where altered gene expression causes embryonic lethality.

## Methods

### Ethics statement

All animals were cared for in accordance with guidelines set forth by the Association for Assessment and Accreditation of Laboratory Animal Care. The University of North Carolina – Chapel Hill's “Institutional Animal Care and Use Committee” (IACUC) approved all studies (protocol #08-045 and #07-228).

### Modifications of the *Ctgf* gene

In order to inactivate the *Ctgf* gene, we have made a targeting construct to delete exons 3 through 5 of the *Ctgf* gene in the genome in ES cells as illustrated in Fig. 1A. The 5′ and 3′ arms of homology are a 4.7 kb BamH1/Nhe1 fragment and a 1.4 kb Nsi1/Bgl2 fragment, respectively, and a neomycin resistant gene (neo) was used as a selection marker. The *Ctgf-Lo* allele (Figure 1B) was made by replacing the endogenous *Ctgf* gene 3′UTR sequence with a cassette that includes a loxP site, followed by the 3′UTR sequence from the *c-Fos* gene (*c-Fos*-3′UTR), neo, a second lox P site, and the 3′UTR from the bovine growth hormone gene (*bGH*-3′UTR). A 1060 bp fragment of DNA including the *Ctgf*-3′UTR sequence, 510 bp of 3′flanking and the polyA addition signal were removed from the endogenous locus leaving the 20 bp immediately following the TAA stop codon intact.

Gene targeting in mouse ES cells (TC1) from 129S6/SvEvTac (129/SvEv) was carried out using the procedures described previously [27], [51]. Cells with planned mutations were identified with PCR, followed by confirmation with Southern blot analyses, and used for generating mice carrying the mutations.

### Mice

Both the *Ctgf KO* and *Ctgf Lo-Hi* mice were established and maintained on a 129S6/SvEvTac (129/SvEv) background. The *Ctgf Lo-Hi* mice were also backcrossed to C57BL6/J (B6) then intercrossed two to three generations to create a mixed B6.129 background. Initial experiments were with these mixed background animals as noted.

The B6.FVB-TgN(EIIa-Cre)C5379Lmgd/J (EIIa-Cre) constitutive global Cre expressing mice were a gift from Dr. Westphal at the NIH [48]. The EIIa-Cre mice were backcrossed to 129/SvEv mice for at least 18 generations before being bred to the *Ctgf Lo-Hi* mice.

The inducible global Cre expressing mice are B6.Cg-Tg(CAG-cre/Esr1)5Amc/J from The Jackson Laboratory, Bar Harbor, ME stock number: 4682 (CAG-Cre) [28]. This Cre line uses the **c**hicken beta **a**ctin promoter/enhancer coupled with a cytome**g**alovirus enhancer (CAG) under control of a mutant mouse estrogen receptor ligand binding domain. The latter receptor ligand domain is unresponsive to estrogen but sensitive to the synthetic ligand 4-hydroxytamoxifen (tamoxifen). Therefore, the CAG-Cre allele produces robust expression of Cre only in the presence of tamoxifen [28].

### Genotyping

Animals were genotyped as appropriate using DNA from either toe or tail snips using standard protocols. *Ctgf KO* animals were genotyped by PCR with the primers: 5′-TCG AGT TCA GAA CCA GAG CT-3′ (common), 5′-TCC GAT TCC TAC CAG GAA GT-3′ (endogenous), and 5′-TTA TGG CGC GCC ATC GAT CT-3′ (neo) with the conditions (92°C for 30 sec., 58°C for 30 sec, 60°C for 7 min) for 35 cycles. Because amplification is passing through the highly repetitive 3′UTR region the amplification step is done at a lower temperature to improve Taq fidelity. The endogenous band is 550 bp and the KO band is 320 bp.

*Ctgf Lo* alleles were genotyped by PCR with the primers: 5′-CAC TCT GCC AGT GGA GTT CA-3′ (common), 5′-TAA TTT CCC TCC CCG GTT AC-3′ (endogenous), and 5′-CAC AGC CTG GTG TGT TTC AC-3′ (*c-Fos*) and the conditions (92°C for 30 sec, 57°C for 45 sec, 65°C for 2.5 min) for 35 cycles. The endogenous band is 525 bp and the *Ctgf KO* band is 375 bp.

Genotyping for *Ctgf Hi* allele was performed using fluorescent primer probe sets in real time PCR with ABI 7500 Fast Real Time PCR 3 system (Life Technologies Carlsbad, CA). The *Ctgf Lo* allele was defined as the presence of the *bGH*-3′UTR and neo and the absence of Cre (where applicable). The *Ctgf Hi* allele was defined as the presence of both the *bGH*-3′UTR and Cre as well as a the absence or low neo gene copy number. Primer sets for *bGH*-3′UTR: TGC CAG CCA TCT GTT GTT TG (forward), ACA GTG GGA GTG GCA TCT T (reverse), and FTC TCC CCC GTG CCT TCC TTG AQ (probe), for neo: GAC GGC GAG GAT CTC GTC G (forward), TAT GTC CTG ATA GCG GTC CG (reverse), and FAC CCA TGG CGA TGC CTG CTT GCC GQ (probe), and for Cre: GGC AGT AAA AAC TAT CCA GCA (forward), GCC GCA TAA CCA GTG AAA CA (reverse), FAT TGC TGT CAC TTG GTC GTG GCA GCQ (probe). For all probes F is 5′ fluorescein (FAM) and Q is the 3′ quencher (TAMRA).

### Quantitation of Neo gene copy number

When genotyping by real time PCR relative gene copy number for the allele of interest, in this case the neo gene, is quantitatively measured. We have used the relative neo gene copy number information from RT-PCR genotyping as a proxy for determining the efficiency of excision of the Lo (*c-fos*-3′UTR and neo) portion of the *Ctgf Lo-Hi* allele.

### Embryo Recovery

To recover embryos, females were checked for mucosal plugs every morning. If a plug was observed the female was separated and watched for pregnancy by visual inspection. At the designated embryonic day point (from E10 – E15 for these experiments) mothers were sacrificed and embryos were recovered live. The embryonic sac (amnion) was separated from each embryo and used for genotyping. Each intact embryo was rinsed in PBS then weighed whole (when possible) and assessed for phenotype. At recovery, embryos were phenotypically classified based on body weight and gross appearance before genotype was determined. “Normal” embryos were nondysmorphic in appearance and at the expected developmental stage for the embryonic age and were within two standard deviations (heavier or lighter) of the mean embryo weight for that litter. “Small” embryos were nondysmorphic in appearance and at the expected developmental stage for the embryonic age, but were small in size and had a body weight below two standard deviations of the litter average (litter averages include all pups that are sufficiently intact to be weighed). “Small atypical” embryos were developmentally delayed, macerated, or poorly vascularized and had a body weight below two standard deviations of the litter average. Embryos were fixed with 4% paraformaldehyde or gluteraldehyde as needed for further studies.

### *Ctgf* Gene Expression Profiling

Mice were euthanized with an overdose of 2,2,2-tribromoethanol and mRNA was harvested from tissues for gene expression profiling. Assays were performed by real-time quantitative RT-PCR as previously described [27]. The primer/probe sets used for *Ctgf* mRNA expression were: AGT CGC CTC TGC ATG GTC A (forward), GCG ATT TTA GGT GTC CGG AT (reverse), and FCC TGC GAA GCT GAC CTG GAG GAA AQ (probe). All samples were normalized to β-actin using primer set: AAG AGC TAT AGA CTG CCT GA (forward), ACG GAT GTC AAC GTC ACA CT (reverse), and FCA CTA TTG GCA ACG AGC GGT TCC GQ (probe). Additional samples for gene expression were either tail samples from 10–12 day old pups or whole embryos harvested from timed matings at embryonic day E10-12 (days post-coitum). For tamoxifen treated animals, three weeks after tamoxifen treatment ear tissue biopsies were taken by small punch on lightly anesthetized (2,2,2-tribromoethanol) mice at 3–4 months of age.

### Immunohistochemistry

Embryos were recovered and sectioned as described above. Sections were deparrafinned then stained with Rabbit anti-CTGF primary antiboday at 1∶100 or 1∶500 dilution (Genetex Irvine, CA) and a anti-Rabbit Alexa Fluor 594 at 1∶200 dilution (Molecular Probes Eugene, OR). Sections were counterstained with DAPI to visualize nuclei. To correct for autofluorescence, for each section there was an adjacent section stained with secondary antibody only. Sections were examined on a Nikon Eclipse 80i and photographned with Nikon DS-QiMc 12mp digital camera and NIS Elements BR 3.0, SP6 (build 539) software.

### Body mass composition and bone density

Using Dual Energy X-ray Absorptiometry (DEXA) we measured body mass composition and bone density. Mice were lightly anesthetized with isofluorane then scanned, excluding the head, using PIXImus DEXA (Lunar Corporation, Madison, WI). Measurements and calculations were performed as previously described [52]. For analysis of DEXA data, males and females were pooled and put in two groups, either *Ctgf Hi/+* or controls (including sibling controls of the genotypes *Ctgf Lo/+;Cre-*, *Ctgf +/+; Cre^+^*, or completely wild type).

### Tamoxifen Treatment

The *Ctgf Lo/Lo* mice were crossed to the tamoxifen inducible CAG-Cre mice to produce *Ctgf Lo/+* and *Ctgf Lo/+; Cre^+^* mice. At six weeks of age mice were treated with tamoxifen at a dose of 3 mg/kg of body weight for four consecutive days [28], [53], [54]. Following treatment the *Ctgf Lo/+; Cre^+^* mice are designated with the genotype *Ctgf Hi/+*. Mice were monitored for any overt phenotypic changes.

### PCR Validation of *Ctgf Lo-Hi* allele

DNA was isolated from skin biopsy samples of animals that carry a single copy of the modified allele and with or without tamoxifen-inducible global Cre transgene. All animals were treated with tamoxifen four months prior to the collection of the samples. The *Ctgf-Lo* was allele amplified with primer1 (5′-ACA GGA AGA TGT ACG GAG AC-3′, corresponding to the sense strand of *Ctgf* exon 5) and primer2 (5′-GCT ACA TCT CTG GAA GAG GT-3′, corresponding to the antisense strand of cFos 3′UTR) and the *Ctgf-Hi* allele amplified with primer1 and primer3 (5′-CAC CTA CTC AGA CAA TGC GA-3′, corresponding to the antisense strand of bGH 3′UTR sequence. PCR products were run on 1.5% agarose gel and stained with ethidium bromide to visual bands.

### Western Blot and Protein Quantitation of Plasma

All plasma samples for western blot and protein quantitation were obtained through retro-orbital or tail vein bleed on anesthetized mice. 0.5 M EDTA was added to whole blood it was spun to obtain plasma. Plasma was used fresh or frozen at −20°C until used. Pre-poured (Nu-Sep iGel) 10% polyacrylamide gels were loaded with 1 µL of plasma mixed with loading buffer. Gels were run, transferred and blotted by standard methods. Membranes were blocked using 10% nonfat milk in tris-buffered saline with Tween (TBST). Primary CTGF antibody (US Biological Rabbit anti mouse-CTGF) was applied overnight 1∶2000 dilution. Secondary (Cal Biochem Goat anti-Rabbit IgG Peroxidase Conjugated) was applied for 3 hours 1∶5000 dilution. Chemiluminescence (Pierce Biotechnology SuperSignal West Pico Chemiluminescent Substrate) was applied per manufacturer instructions. Band density was quantitated with ImageJ software.

### Microscopy/Photography

Adult mice were lightly anesthetized with 2,2,2-tribromoethanol then photographed live (Canon Powershot SD600, Canon, NY, USA). Whole fixed E13.5 embryos were pictured using a dissecting macroscope (Leica Wild M420 Leica, NY) with a QImaging camera (QImaging MicroPublisher MP3, Surrey, BC, Canada). Whole unfixed E10.5 embryos were pictured with embryos immersed in buffered saline using light microscopy (Olympus America Inc., Melville, New York). Embryos were then fixed and prepared for scanning electron microscopy (EM) as previously described [55]. Briefly, embryos were fixed in 2.5% glutaraldehyde in Sorenson's buffer. Secondary fixation was performed in 2% osmium tetroxide in Sorenson's buffer [55]. Samples were dehydrated in consecutive ethanol washes then dried. Specimens were mounted on aluminum stubs with colloidal silver, sputter coated, then viewed and photographed by scanning EM (Zeiss Supra 25 field emission scanning electron microscope, Carl Zeiss, Thornwood, NY, USA).

### Statistics

Data are presented as mean ± standard error of the mean and p-values are from paired Student t-test performed in Microsoft Excel. Additional statistics including Chi Squares were performed by hand. Any p-value less than 0.05 was considered statistically significant.
